# SuperPath approach is a recommendable option in frail patients with femoral neck fractures: a case–control study

**DOI:** 10.1007/s00402-021-04153-y

**Published:** 2021-09-04

**Authors:** Antonio Benedetto Cecere, Annalisa De Cicco, Gaetano Bruno, Giuseppe Toro, Giacomo Errico, Adriano Braile, Alfredo Schiavone Panni

**Affiliations:** 1 Department of Medical and Surgical Specialties and Dentistry, University of Campania “Luigi Vanvitelli”, Via L. De Crecchio 4, 80138 Naples, Italy; 2 Unit of Orthopaedics and Traumatology, AORN Sant’Anna E San Sebastiano, Caserta, Italy

**Keywords:** Hip fracture, Blood loss, Blood transfusion, Surgical approach, SuperPath, Hemiarthroplasty

## Abstract

**Introduction:**

The treatment of intracapsular femoral neck fractures (FNFs) in the elderly is usually based on hip replacement, both total hip arthroplasty (THA) and hemiarthroplasty (HA). Recently, several tissue-sparing approaches for hip arthroplasty had been described with promising results in terms of hospitalization length, blood loss and dislocation rate. The aim of the present study was to compare the blood loss and the transfusion rate in a cohort of patients with FNF treated using an HA through both the SuperPath (SP) and the traditional posterolateral (PL) approaches.

**Materials and methods:**

We retrospectively collected data from patients affected by FNFs between January 2018 and February 2020. All patients with intracapsular FNF treated with a single HA implant (Profemur L, MicroPort Orthopedics Inc., USA) via PL or SP approaches were included. Exclusion criteria were pathological fractures, polytrauma and preoperatively transfused patients.

**Results:**

Thirty-five patients were included and analysed in the present study. 17 patients were classified in the SP group, and 18 in the PL one. The rate of antithrombotic therapy was higher in the SP group compared with the PL group [10 (58, 82%) vs 4 (22, 2%)]. While the two groups did not differ in terms of preoperative haemoglobin (Hb), 48 h postoperative Hb and Hb reduction, a significative difference was observed in terms of blood transfusion rate (1 SP vs 9 PL, *p* = 0.0072).

**Conclusions:**

The SuperPath approach in patients with FNF under antithrombotic therapy assures lower transfusion rate, potentially reducing complication rates and improving patients' outcomes.

## Introduction

The treatment of intracapsular femoral neck fractures (FNFs) in the elderly is usually based on hip replacement, both total hip arthroplasty (THA) and hemiarthroplasty (HA). The choice between these two techniques is still debated. HA is generally preferred in older patients, because of lower surgical time, blood loss and dislocation rate [1–3]. Moreover, the risk of early revision due to acetabular wear related to HA could be considered irrelevant in the very elderly, considering their low functional demand [4].

Recently, several tissue-sparing approaches for hip arthroplasty have been described with promising results in terms of hospitalization length, blood loss and dislocation rate [5–7]. The SuperPath (SP) approach is a posterior tissue-sparing hip approach, already successfully used to treat FNFs in the elderly [8]. Also in this population early mobilization, preserved gait kinematics and pain reduction could be achievable using tissue-sparing approaches [8].

Moreover, compared to the traditional posterolateral (PL) approach, the SP, preserving short external rotator tendons and the posterior capsule, further reduces the blood loss and the risk of hip dislocation. In frail patients, even a slight reduction of haemoglobin might be life threatening, and therefore a high blood transfusion rate was reported in patients with FNF [9]. However, blood transfusions are not risk free and significantly impact the healthcare system in terms of both direct and indirect costs. In fact, a higher risk of early morbidity and mortality among patients with geriatric hip fracture was observed [9]. Some specific complications were reported with blood transfusions, including transfusion-related lung injury (TRALI) and multi-organ dysfunction, due to a decrease in the red cell ability of oxygen delivery [10–12]. Additionally, although in the last few years the risk of infection transmission and lethal transfusion reactions had been constantly decreased, the incidence of any type of transfusion reaction is still consistent. In fact, Castillo et al. estimated an incidence of transfusion-related reactions in up to 1% of patients [12].

The aim of the present study was to compare the blood loss and the transfusion rate in a cohort of patients with FNF treated using an HA through both the SP and the traditional PL approaches.

Our hypothesis was that the use of the SP approach would be able to reduce blood loss and blood transfusion rate in a population of elderly with fragility FNF.

## Materials and methods

We retrospectively collected data from patients affected by FNFs between January 2018 and February 2020. All patients with intracapsular FNF treated with a single HA implant (Profemur L, MicroPort Orthopedics Inc, USA) via PL or SP approaches were included. Exclusion criteria were pathological fractures, polytrauma and preoperatively transfused patients.

We collected demographic data, length of hospitalization, comorbidities, length of surgical procedure, transfusion rate, and haemoglobin (HB) values preoperatively and 48 h after surgery. The amount of blood loss was evaluated through Delta HB values, defined as the difference between preoperative and 48 h postoperative haemoglobin values.

Depending on the surgical approach used, patients were divided into two groups: SuperPath (SP) and posterolateral (PL) group. In all cases the prosthesis was implanted in the lateral decubitus over the unaffected side. In the SP approach, the affected hip was positioned at 45° of flexion and 10–15° of internal rotation. A 6–8 cm skin incision was performed taking the greater trochanter as a reference. After opening the gluteal fascia, the gluteus maximus was separated and the space between the gluteus minimus and the piriformis was exposed to access the capsule. The joint tendons or external rotator muscles were not excised. In the PL group, the skin incision begins posterior to the lateral side of the greater trochanter and runs distally about 6 cm along the femoral axis. Proximally, the incision runs slightly curved towards the PSIS to a point approximately 6 cm proximal to the greater trochanter. The fibres of the gluteus maximus were dissected and the greater trochanter was visible. The external rotators were released and retracted medially, and the capsule was exposed.

To identify differences between groups, descriptive statistics, Student’s *T* test and Fisher exact tests were used. Significance was set at *p* < 0.05.

All patients provided written and informed consent allowing to undergo surgery and to have their data collected for scientific and audit purposes, as a standard protocol. The present study has been performed in accordance with the ethical standards as laid down in the 1964 Declaration of Helsinki and its later amendments or comparable ethical standards. According to Italian law, formal ethics approval was not required for this study, as it involved routine tests and patient evaluations.

## Results

**Fig. 1:**
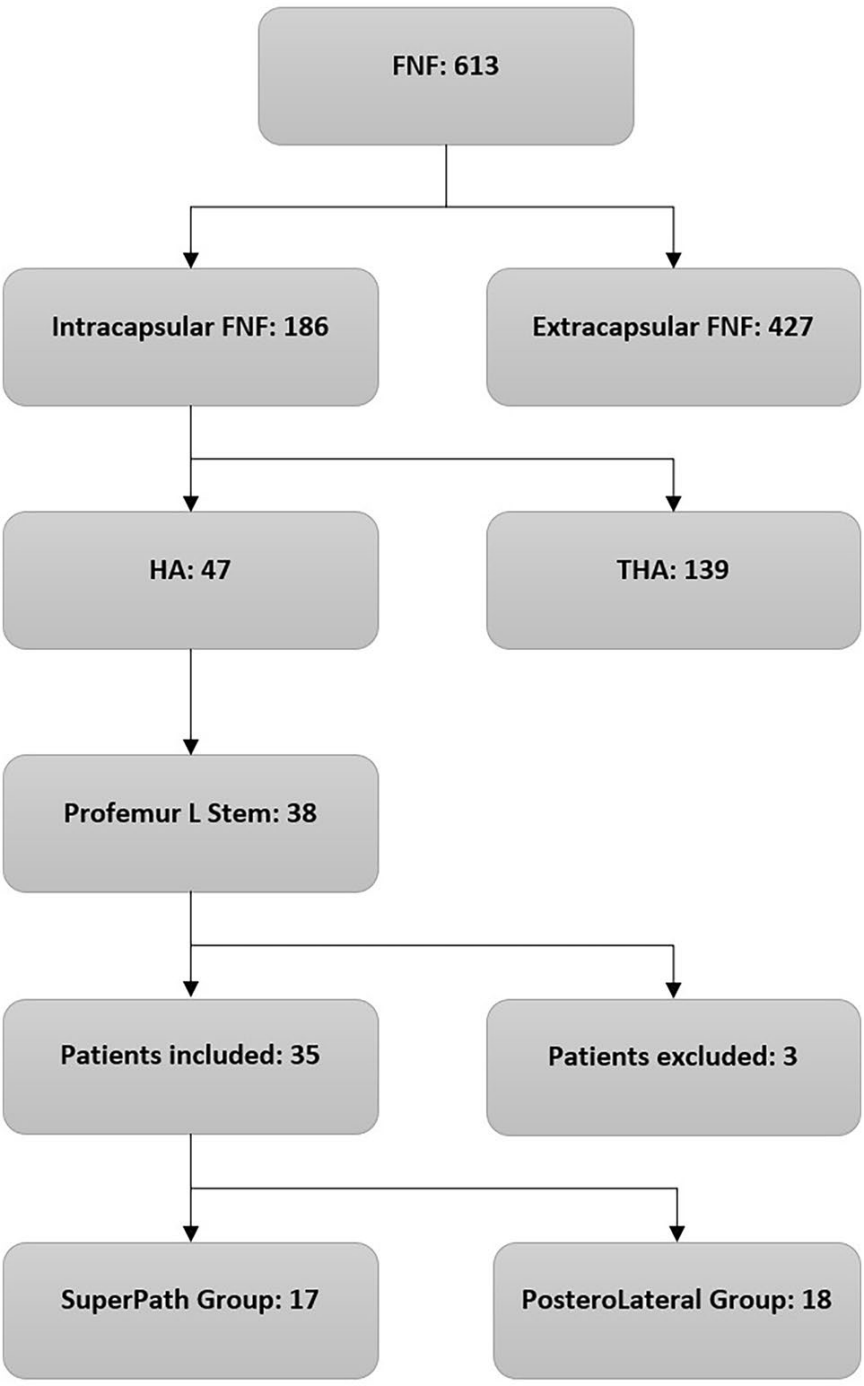
Patients’ selection process

During the inclusion period, 613 patients with an FNF were admitted to our institution, 186 of them were affected by an intracapsular one. Forty-seven intracapsular fractures were treated with HA. Among these, 38 Profemur L stems were implanted. Three of 38 patients were excluded, 1 because of pathological fracture and 2 because of receiving a blood transfusion prior to surgery. Therefore, 35 were included and analysed in the present study. 17 patients were classified in the SP group, whereas 18 in the PL one (see Fig. 1).

Patients’ data and group distribution are shown in Table 1.

**Table 1:** Characteristics of the study groups

	SP group	PL group	*p* value
Total number	17	18	n.a
Sex	3 m, 14f	3 m, 15f	1
Age (SD)	84*y* (*σ* 7, 45)	85, 1*y* (*σ* 7, 72)	0.667
Length of hospitalization days (SD)	12, 65 (*σ* 4, 65)	13, 83 (*σ* 3, 71)	0.206
Chronic atrial fibrillation (%)	5 (29, 4%)	2 (11, 1%)	0.2285
Hypertensive cardiomyopathy (%)	5 (29, 4%)	6 (33%)	1
Chronic heart failure (%)	3 (17, 6%)	1 (5, 55%)	0.3377
DM type 2 (%)	2 (11, 7%)	1 (5, 55%)	0.6026
Senile dementia (%)	1 (5, 9%)	2 (11, 1%)	1
Epilepsy (%)	1 (5, 9%)	0 (0%)	0.4857
No comorbidity (%)	3 (17, 6%)	6 (33%)	0.4430
Antithrombotic therapy (%)	10 (58, 82%)	4 (22, 2%)	0.0799
Surgical time min (SD)	82,2 (*σ* 37, 42)	81, 39 (*σ* 48, 71)	0.458
Transfusions (%)	1 (5, 88%)	9 (50%)	0.0072

*SP* SuperPath, *PL* posterolateral

The two groups did not differ in age (mean 84 SP vs 85.1 PL), sex distribution (M:F ratio: 1:4.6 SP vs 1:5 PL), length of surgery (minutes: 82.2 SP vs 81.39 in PL) and length of hospitalization (days: 12.65 SP vs 13.83 PL).

Comorbidities were more commonly represented by chronic hypertensive cardiomyopathy (HC), chronic atrial fibrillation (CAFib), chronic heart failure (CHF) and type 2 diabetes mellitus (DM type 2) in both groups. The rate of antithrombotic therapy was higher in the SP group compared with the PL group [10 (58, 82%) vs 4 (22, 2%), *p* value 0.0799].

Haemoglobin values are shown in Table 2. The groups did not differ in terms of preoperative Hb (12.33 mg/dL SP VS 12.29 mg/dL PL), 48 h postoperative (10, 04 mg/dL SP VS 9.81 mg/dL PL) and DELTA Hb (2.29 mg/dL SP VS 2.48 mg/dL) values.

**Table 2:** Haemoglobin value distribution in the studied groups

HB values (mg/dl)	SP group (mean, SD)	PL group (mean, SD)	*p* value
Preoperative HB	12, 33 (*σ* 1, 31)	12, 29 (*σ* 1, 69)	0, 936, 984, 825
48 h postoperative Hb	10, 04 (*σ* 1, 06)	9, 81 (*σ* 1, 94)	0, 664, 722, 034
Hb Delta	2, 29 (*σ* 1, 01)	2, 48 (σ 1, 41)	0, 640, 803, 887

*SP* SuperPath, *PL* posterolateral, *HB* haemoglobin

The two groups significantly differed in terms of blood transfusion rate (1 SP vs 9 PL, *p* = 0.0072).

## Discussion

FNFs are considered as one of the most relevant health problems that the Western civilization has to face, considering the constant increase in terms of incidence, costs [13, 14] and a 1-year mortality rate of up to 33% [15]. FNFs are generally classified in extracapsular and intracapsular. These two types of fractures widely differ in terms of management, and patients’ characteristics and outcomes [16]. Intracapsular FNFs in the elderly are generally treated using both THA and HA. These latter are associated with lower surgical time and dislocation rate [17], representing thus a viable option especially in the very elderly.

THA in this population are associated with a high risk of life-threatening complications including dislocations, thromboembolism, infections and periprosthetic fractures (PPF) [16, 18–20]. One of the most relevant factors that could influence the outcomes of the THA in the elderly with FNF is the poor bone quality [21]. The surgical approach could influence the frequency of some complications. In example, it was observed that both the lateral and the anterolateral approaches were associated with a lower dislocation rate compared to the PL [3]. Minimally invasive approaches present the supposed benefits of minimizing damage to surrounding soft tissues, with lower dislocation rates, reduced pain, early mobilization and lower hospitalization [22].

However, FNF bad outcomes are more frequent in nursing home patients with multiple comorbidities. For this reason, the use of tissue-sparing techniques would be useful in this population to improve rehabilitation and prevent complications. Pulmonary infections, deep vein thrombosis, muscular dystrophy and pressure ulcers are the main comorbidities related to long-term immobilization and postoperative pain [23].

The present study represents one of the few reports that analysed the reliability of the SP approach for the treatment of FNF in the elderly [8, 24, 25].

Our findings were similar to those reported by Xu et al.[24], which did not find a difference in total blood loss between the two groups analyzed (HA via SP VS HA via PL approach).

The most relevant finding of our study was the lower blood transfusion rate in the SP group despite the higher percentage of patients under antithrombotic drugs. This latter, in fact exposes patients to a greater risk of intra- and postoperative blood loss and, therefore, to a greater risk of transfusions [26].

Blood transfusions are associated with multiple disadvantages, such as prolonging the hospital stay, transfusion reactions, surgical site infections, and thrombotic and cardiac events [27, 28]. Blood transfusions represent a relevant economic burden for healthcare systems.

Several studies estimated the cost of blood transfusion in the USA to be from $ 515.63 to $ 1303.68 [29–31]. On the other hand, a recent systematic review estimated the cost of 2 unit blood transfusion in Western Europe to be approximately € 878. 21 [32].

Most of the transfusion reactions are mild and easily resolvable, but could be also extremely severe and fatal [18].

Our findings were supported by those reported by Xu et al., Bodrogi et al. and Jianbo et al. [8, 25].

Particularly Jianbo et al., comparing SP vs PL approaches in hip hemiarthroplasty in elderly patients, found a tripled transfusion rate in the PL group [8].

Interestingly, our transfusion rate (5.88% in SP group) was even lower than that reported by Bodrogi et al. and Xu et al. (23.5 and 26.6% respectively) [24, 25]. Table 3 summarizes the available knowledge of the transfusion rate and blood loss using the SP approach in FNF.

**Table 3:** Comparison between the present study and the available literature in transfusion rate and HB reduction

Transfusion rate	SP group	PL group	*p* value
Jianbo 2019 [8]	4.0% (2/50)	18% (9/50)	0.025
Xu 2019 [24]	26.9% (14/52)	35.9% (28/78)	
Present study	5.88% (1/17)	50% (9/18)	0.0072

Hb delta values	SP group	PL group	*p* value
Xu 2019 [24] (72 h postop)	2.67 ± 1.79	2.48 ± 1.51	
Present study (48 h postop)	2.29 (± 1.01)	2.48 (± 1.41)	0.664722034

SP SuperPath, *PL* posterolateral, *HB* haemoglobin

In our opinion, these observations support the use of SP in the elderly with FNF under antithrombotic therapy. In fact, lowering the transfusion rate might be associated with a reduction of wound infection [33] and healthcare costs [34].

Our study has some limitations. The retrospective nature of the study and the absence of a randomization might lead to allocation bias, and the small sample size to an underpowered statistical analysis. However, the two groups were similar in terms of age, sex and comorbidities. Furthermore, the use of strict inclusion criteria (i.e. the use of a single type of prosthesis) allowed us to reduce confounding variables.

## Conclusions

The present study represents one of the few reports that analysed the reliability of the SP approach for the FNF in the elderly. This tissue-sparing approach seems to be effective in reducing the rate of transfusions. This data could positively affect a rapid functional recovery, thus reducing complications due to bed rest and health costs. Considering these results, the use of tissue-sparing approaches in patients with FNF under antithrombotic therapy may be desirable. However, a randomized controlled trial may be advisable to confirm our data.

## Data Availability

Data and materials included in the present study are available upon reasonable request.
